# Assessment of the levels of termination of the conus medullaris and thecal sac in the pediatric population

**DOI:** 10.1007/s00234-022-03111-8

**Published:** 2023-01-21

**Authors:** Mohammed Blaaza, Carolina Filipa Chaves Figueira, Mohamed Rashad Ramali, Cillian McNamara, Mariasavina Severino, Domenico Tortora, Kshitij Mankad, Andrea Rossi

**Affiliations:** 1 University College London Medical School, London, UK; 2 Neuroradiology Unit, Hospital Central do Funchal – SESARAM, Madeira, Portugal; 3 Great Ormond Street Hospital Radiology, London, UK; 4 Neuroradiology Unit, IRCCS Insituto Giannina Gaslini, Genoa, Italy; 5 Department of Health Sciences (DISSAL), University of Genoa, Genoa, Italy

**Keywords:** Conus medullaris, Thecal sac, Conus termination, Pediatric spine

## Abstract

**Purpose:**

This study assessed the position of the termination of the conus medullaris (the point where the spinal cord tapers to an end) and thecal sac (the sheath of dura mater that surrounds the spinal cord and caudal nerve roots) in a large pediatric population, to characterise the nature of the pediatric Gaussian distribution and assess whether age affected the distribution. The study further aimed to assess the effect of gender on termination positions.

**Methods:**

A total of 520 MRI spine studies of children aged between 1 month and 19 years old were collected from two pediatric tertiary referral centres in the UK and Italy. Studies with pathological findings were excluded, and normal scans were found using keyword search algorithms on a database of radiologists’ reports. The reported scans were individually assessed and reviewed by two experienced neuroradiologists. The termination points of the conus medullaris and thecal sac were determined for each study. Local IRB approvals were sought.

**Results:**

The results showcased a Gaussian distribution in both conus medullaris (*r*=0.8997) and thecal sac termination levels (*r*=0.9639). No statistically significant results were noted with increasing age for the termination positions of the conus medullaris or thecal sac (*p* = 0.154, 0.063). No statistical significance was observed with gender variation with either anatomical landmark. A weak positive correlation was observed between the termination levels of the conus medullaris and the thecal sac (*r*=0.2567)

**Conclusion:**

Termination levels across all pediatric age range followed a Gaussian distribution. Knowledge of normal termination levels has relevant clinical implications, including the assessment of patients with suspected spinal dysraphism.

**Supplementary Information:**

The online version contains supplementary material available at 10.1007/s00234-022-03111-8.

## Introduction

Embryologically, the spinal cord forms mostly from the neural plate generated during primary neurulation, with only its caudal metameres (i.e., S3-S5 and coccygeal levels) and filum terminale deriving from the processes of junctional and secondary neurulation [1]. The conus medullaris (CM) is the point where the spinal cord tapers and comes to an end. During fetal development, the CM progressively “ascends” along the vertebral column as a combined result of the phenomenon of retrogressive differentiation of the secondary neural tube and the differential longitudinal growth rates of the vertebral column and spinal cord. The CM eventually occupies its final, “adult” position shortly after birth, but studies have differed on the precise time at which this is reached, i.e., whether the conus settles in its final position at 2 months or at 5 months of age [2]. In fact, some of the literature has suggested that no further ascent occurs after birth and that the ascent entirely occurs within gestation, especially between weeks 9 and 16 [3]. Thus, this study sought to further clarify the matter and to provide statistically significant conclusions on the timeframe when the final position is achieved.

Although the variation of its position is found among individuals, its peak incidence in the adult population has been reported at the level of the L1 vertebra [4], with a mean variation ranging from the T12 to the upper L3 vertebrae. Similarly, slight variation is seen in the adult position of the thecal sac terminus (TS); however, TS is generally expected to terminate at the level of the S2 vertebra, with mean variation ranging from the lower L5 to the lower S3 vertebrae [4, 5].

Several studies have long established a Gaussian distribution in the termination levels of the conus medullaris and the thecal sac in the adult population. However, there lies existing variation in the literature as to what age the adult termination position is achieved. Thus, this study aimed to characterise the pediatric Gaussian distribution further and assess whether any changes existed across the age range.

Few studies have been conducted solely in the pediatric age groups, and there is yet to be sufficient literature ascertaining whether changes in the Gaussian distribution occur across increasing pediatric age and gender. This study, with its large sample size (*n*=520) aimed to reinforce this distribution amongst pediatric populations.

Prior studies [6] have employed similar methodologies to assess at which age in particular the adult termination level is attained. This study sought to further quantitatively outline the nature of the Gaussian distribution itself, and ascertain whether age or gender had an important role to play in this respect. Previous literature [7] has assessed the effect of gender of distribution, however, was limited by an upper range of 6 months of age, as opposed to the entire pediatric age range. In addition, this study only considered ultrasound as the imaging modality, which has its own limitations.

## Methods and materials

This study was a retrospective observational study conducted in two tertiary referral centres in the UK and Italy with local IRB approvals. The selected age range of subjects was 1 month to 19 years (mean age 7.78 years). The inclusion criteria included subjects with normal MRI whole spine. We included patients with brain tumours and cranial trauma with normal spinal imaging. Patients with spinal dysraphism, vertebral segmentation anomalies, those with previous spinal cord disease or injury, or, those who had undergone previous spinal surgery for whatever reason were excluded.

The patient databases were narrowed down using these inclusion and exclusion criteria, and the relevant MRI studies were reviewed. Each data point was subsequently manually reported by two experienced neuroradiologists. The sequences were acquired using 1.5T scanners as part of routine clinical protocols which included T1, T2 axial, and sagittal sequences and/or Short-TI Inversion Recovery (STIR). The images were taken in the supine position as per standard clinical practice. Termination level was selected at the level where the tapering of the conus and thecal sac could be seen most clearly, with all slices considered before the optimal slice selection.

The selection process included reviewing patient databases on tens of thousands of imaging reports and restricting our search to only those where an experienced neuroradiologist had previously reported that the spine appeared normal, or similar words to that effect. This technique excluded several thousand cases, with the remaining cases assessed chronologically, until an adequate sample size was obtained. Studies with unclear quality were excluded, due to the possibility of bias.

There was no assessment for interrater agreement for the measurements; however, in equivocal cases, a joint opinion between two experienced neuroradiologists was agreed upon

**Fig. 1:**
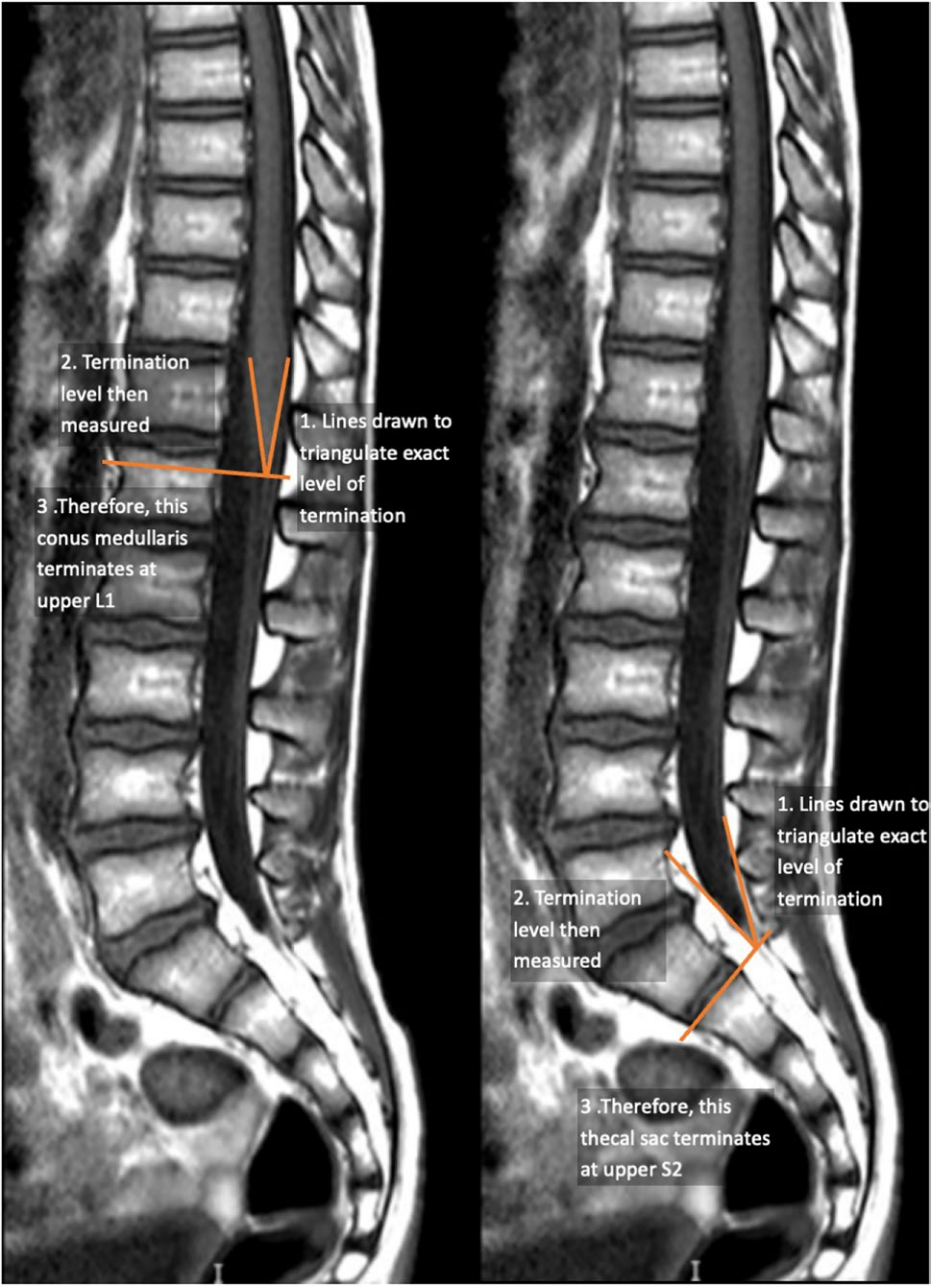
A showcase of how the level of termination of the thecal sac and the conus medullaris were measured in steps 1–3

The images were analysed in the sagittal plane, and the same technique was used to measure the level of both terminations of the CM and TS. Lines were drawn to triangulate the exact point at which each structure terminated so that the corresponding vertebral level could be ascertained by drawing a perpendicular line from the termination point. The vertebral level was found by counting from top downwards and upwards from the last lumbar vertebra, which was identified as the last well-formed vertebral body above the sacrum. Fig. 1 shows the approach to assessing the level of termination.

In accordance with techniques adopted in similar previous studies, the specific vertebra where the structures terminated was then divided into thirds (upper, middle, and lower) so that the level of termination could be ascribed to a more specific part of the vertebra. In cases where they terminated at an intervertebral disc, they were labelled as being at the level of the appropriate disc. Finally, to conduct statistical analysis, each vertebral level was allocated a numerical value. For the termination of the conus medullaris, it ranged from 1 (lower third of T11) to 16 (middle third of L3), and for thecal sac termination from 1 (L5-S1 disc) to 13 (S3-S4 disc).

### Statistical analysis

The Product Moment Correlation Coefficient was used to describe the normality of the data, whereby our obtained dataset was contrasted to a perfectly generated counterpart using its key parameters. This technique has been employed in previous studies. We further calculated kurtosis and skewness values, to reinforce the Gaussian distribution.

The effect of gender on the level of termination was assessed using the Wilcoxon signed rank test. The effect of age on the termination levels was assessed using the Kruskal−Wallis ANOVA test. The Product Moment Correlation Coefficient was used to assess for any correlation between the termination levels of the conus medullaris and the thecal sac.

The statistical analysis was conducted and cross-checked internally, by researchers with backgrounds in statistics.

## Materials

This study comprised 520 children across two tertiary referral centres, with an age breakdown denoted in the below tables. The study aimed to assess a sufficient number of cases across each age group, with special emphasis on those in the first year of life, assessing 103 individuals. Relatively few imaging studies were available in the first month of life across both centres.

**Table:** 

**Age in months**	**Frequency**
0m	1
1m	6
2m	3
3m	7
4m	5
5m	12
6m	9
7m	4
8m	6
9m	3
10m	13
11m	2
12m	32

**Table:** 

**Age in years**	**Frequency**
2	28
3	41
4	30
5	22
6	21
7	26
8	22
9	33
10	37
11	30
12	23
13	22
14	17
15	29
16	15
17	15
18	6

## Results

A total of 520 MRI studies were assessed from the two centres (250 females and 270 males).

### Level of termination of the conus medullaris

**Fig. 2:**
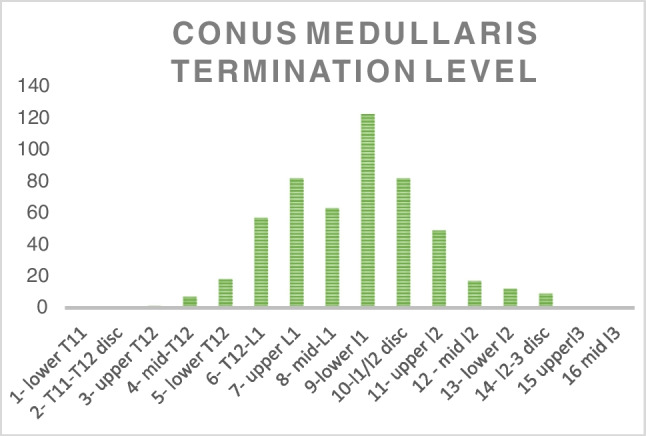
Graph showing the distribution of conus medullaris termination levels in the patient population. Termination position is shown on the x-axis, and the number of studies terminating at each level is shown on the y-axis

A Gaussian distribution to the level of the conus termination was found, with the mean at the level of lower L1. When the data was contrasted to that of its perfectly normally distributed counterpart data, an extremely strong positive correlation (*r*=0.8997) was produced (Fig. 2). The population mean was calculated to be 7.904 (95% CI 7.71 to 8.09). The termination levels ranged from upper T12 to the L2/L3 disc.

### Effect of gender on termination level of conus medullaris

The study showed Gaussian distributions in both female and male subset populations. In males, the data for the conus medullaris showed a strong positive correlation against its normally distributed dataset (*r*= 0.9717). Within the female pediatric population, the data was also shown to be normally distributed for the conus medullaris (*r*=0.8942). The mean level of termination of the conus medullaris was 7.88 in males (95% CI 7.74 to 8.04) and 7.93 (95% CI 7.78 to 8.11) in females, both corresponding to the mid-L1 level. The termination level of the conus ranged from upper T12 to the L2-L3 disc in males, whereas it ranged from upper T12 to the mid-L3 vertebrae in females. This is all strongly in keeping with Gaussian distributions across both genders.

**Fig. 3:**
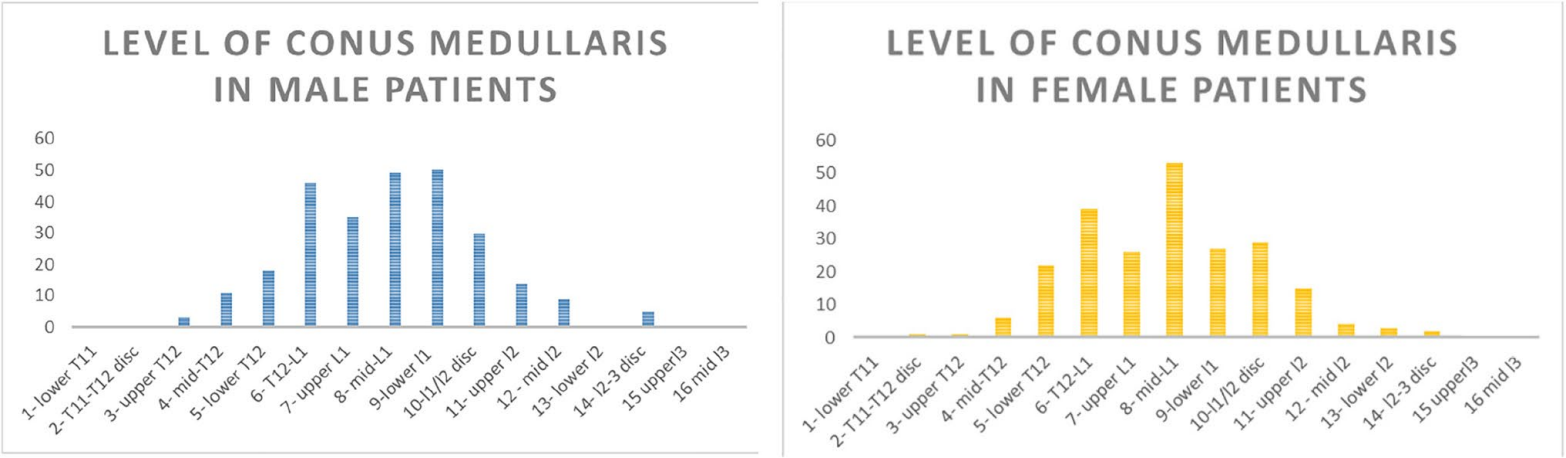
Graph showing the distribution of termination levels of the conus medullaris in male and female pediatric patients. Termination position is shown on the x-axis, and the number of studies terminating at each level is shown on the y-axis

A similar Gaussian distribution was noted for the level of the tip of the thecal sac (*r*=0.9639). Fig. 3 showcases the results, with a Gaussian distribution clearly visualised. The mean level of termination was 7.128 (95% CI (6.95, 7.31)), corresponding to mid-S2. The data ranged from the L5-S1 disc to the S3-S4 disc.

### Effect of gender on termination levels of conus medullaris and thecal sac

**Fig. 4:**
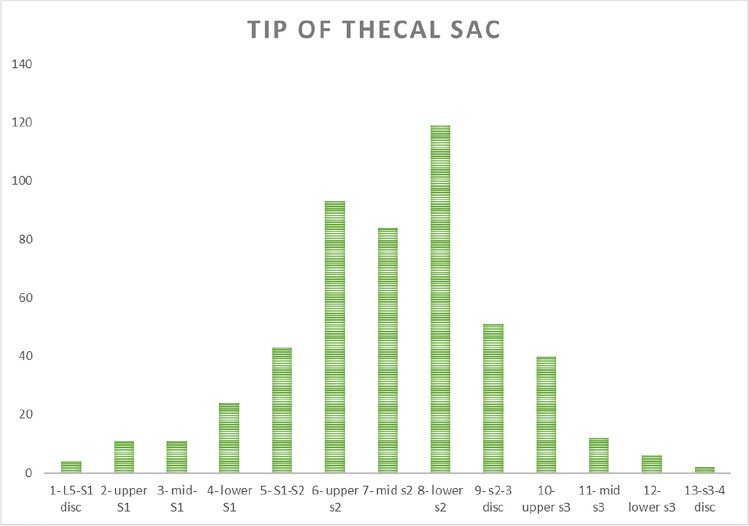
Graph showing the distribution of thecal sac termination levels in the patient population. Termination position is shown on the x-axis, and the number of studies terminating at each level is shown on the y-axis

**Fig. 5:**
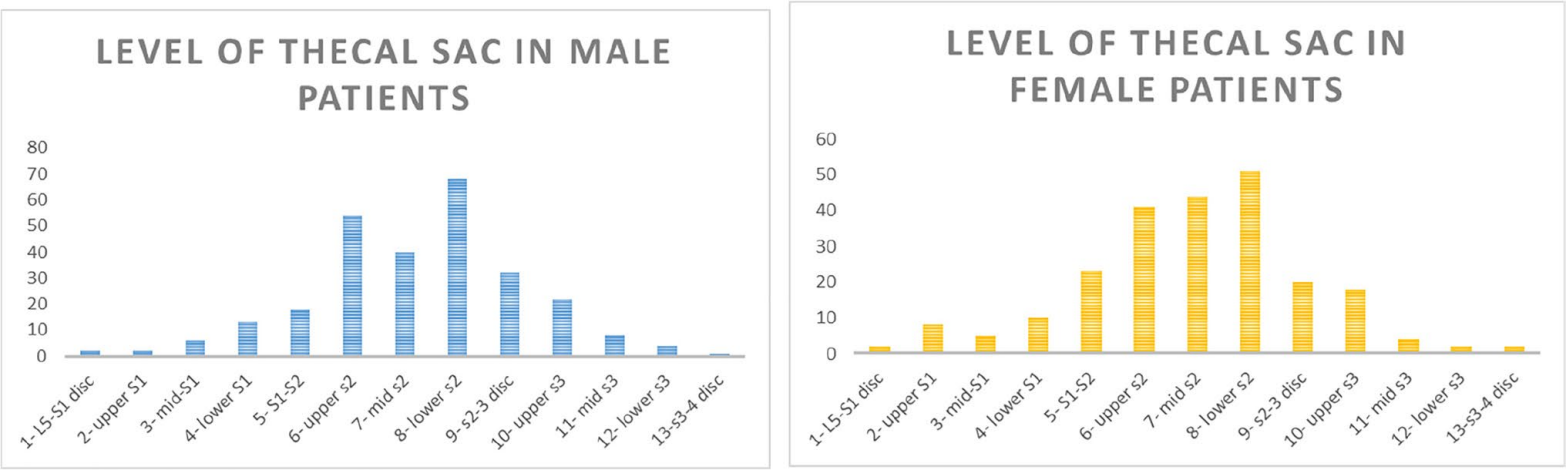
Graph showing the distribution of termination levels of the thecal sac in male and female pediatric patients. Termination position is shown on the x-axis, and the number of studies terminating at each level is shown on the y-axis

The thecal sac data, classified by gender, also showcased a strong Gaussian distribution. The male data was shown to have a strong positive correlation (*r*= 0.9397). The mean level of termination of the thecal sac was 7.28 (95% CI 7.15, 7.43) in males and 6.94 (95% CI 6.78, 7.10) in females, both at the level of lower S2 (Figs. 4 and 5). The data ranged from the L5-S1 disc to the level of lower S3 in males, and the L5-S1 disc to the S3-S4 disc in females.

A 2-tailed Wilcoxon rank sum test was performed and did not allow rejection of the null hypothesis at the 5% significance level (*p*=0.9955). This showed no statistically significant difference between the distribution of termination positions in the male and female populations. This strengthens the previously published view that gender has no noteworthy impact on the positions of termination.

### Correlation between the position of conus medullaris termination and thecal sac termination

**Fig. 6:**
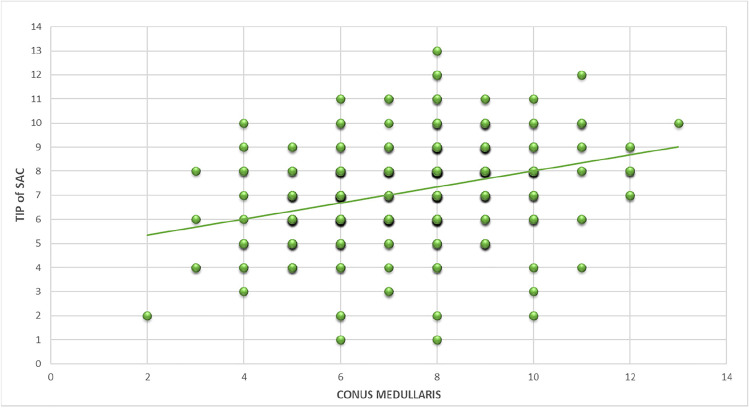
Graph showing the correlation between the levels of termination of the conus medullaris and thecal sac. Termination levels of conus medullaris are shown on the x-axis and that of the thecal sac on the y-axis

A positive correlation (*r*=0.2567) was noted between the levels of termination of the conus medullaris and thecal sac in each patient respectively (Fig. 6). This is in line with the published data in adults (5).

### Effect of age on the levels of termination of the conus medullaris and the thecal sac

**Fig. 7:**
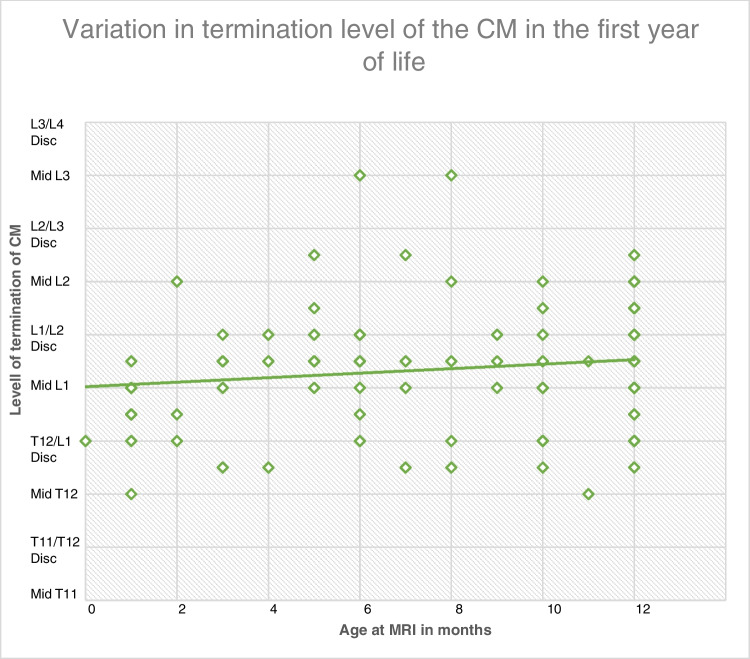
Graph showing the effect of age on the distributions of termination level of the conus medullaris in the first year of life. Age in months is shown on the x-axis, and the termination position is shown on the y-axis.

The data across pediatric age ranges was compared for any trends. Firstly, the subset of the first 6 months of life was considered in isolation to further elucidate whether a clear ascent was noted within this time frame (Fig. 7). The graph shows no noteworthy changes in termination level across age in the first year of life. A Kruskal-Wallis ANOVA test was performed to assess whether age had a significant impact on the level of termination of the conus medullaris in the first 6 months of life. A non-significant *p* value of 0.1218 was obtained, suggesting no ascent occurs in the first 6 months.

The entire pediatric age range was subsequently considered and is shown in supplementary figure 1. The graph shows no noteworthy changes in termination levels across age in the pediatric population. A Kruskal-Wallis ANOVA test was performed to assess whether age had a significant impact on the level of termination of the conus medullaris in the age range of 0–19 years. A non-significant *p* value of 0.1543 was obtained, suggesting that age has no significant influence on the termination level of the conus medullaris.

**Fig. 8:**
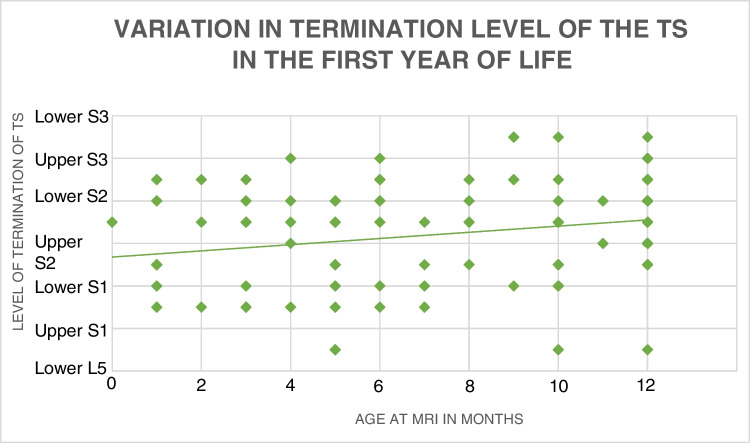
Graph showing the effect of age on the distributions of termination level of the thecal sac in the first year of life. Age in months is shown on the x-axis, and termination position is shown on the y-axis

Similar methods were used to assess the termination positions of the thecal sac in the first year of life and across the pediatric age range respectively. The Kruskal-Wallis analysis obtained *p* values of 0.2859 and 0.063 respectively, suggesting that age has no significant impact on the termination level of the thecal sac. Fig. 8 shows the change in the termination positions of the thecal sac in the first year of life.

## Discussion

This study has several important clinical implications. Irradiation for spinal tumours is traditionally performed by placing the caudal border of the spinal field at S2/S3 intervertebral space [8]. However, such a placement has been shown to miss 8.7% of thecal sacs [8], risking a failure of successful treatment of the underlying tumour. Whilst contouring and placement are continually improving, significant variability is still being observed amongst high-volume practitioners [9]. The clinical risk of underestimating the contour may lead to greater rates of central neuropathy [9]. This serves to denote the extreme clinical importance of a correct understanding of the distribution of the thecal sac and likely termination position in children.

Accurate knowledge of the normal position of the conus is imperative for a correct evaluation of patients suspected of harboiring a spinal dysraphism. In fact, low positioning of the conus is one of the features that neurosurgeons consider when selecting patients who are candidates for detethering surgery.

### Level of termination of conus medullaris

The mean level of termination of the conus in populations of adults was noted to be at upper L1 [4, 10]. This study found a mean pediatric termination around mid-L1 level. This may suggest the conus medullaris is more low-lying in pediatric populations; however, no statistical significance was noted.

This study has concluded that the absolute lower limit for a normal conus medullaris is at the level of the L2-L3 disc. This is in line with previous literature [4]. The current ISPN position is that any conus below mid-L2 should be considered tethered until proven otherwise [11]. This may suggest a new definition of the absolute lower limit of the CM is warranted

### Level of termination of the thecal sac

Once again, these results support the previously published literature on the level of termination of the tip of the thecal sac in adult populations [4]. Similar mean positions were noted, with the tip lying around the level of mid-S2. Previous literature in pediatric populations has concluded an average position of upper S2 [8], although amongst a small sample size (*n*=23). The results are clinically valuable as they may suggest no specific age adjustment needs to be made with respect to the positioning of spinal fields in irradiation procedures, as there is no increased risk of overestimating or underestimating contours.

### Correlation between the position of conus medullaris termination and thecal sac termination

The resultant correlation (*r*=0.2567) is in line with previous studies which have shown a correlation in adult populations to be *r*=0.309 and 0.32 respectively [4, 12]. This allows extrapolation that a similar correlation exists and is maintained throughout all ages. This correlation is unlikely to be due to the result of error, due to both its replicability from previous studies [4] and the relatively large sample sizes in the studies.

Accurate knowledge of the relationship between the CM and TS terminuses can be very important in the assessment of patients with suspected cord tethering who have a conus in a normal position. That is, the overall position of the conus may still be “normal” in general terms (e.g., L2), but the conus is actually tethered and stretched. In this case, there could be a loss of proportionality between the CM and TS. In the reverse cases, i.e. in patients with caudal regression syndrome type I, both the cord terminus and TS are typically higher than normal. Thus, this reinforces the need to assess for proportionality between the CM and TS positions, that is, assisting in the evaluation of the aforementioned subtle cases.

### Effect of gender on position of conus medullaris termination and thecal sac termination

In both male and female children, the mean level of the conus medullaris and thecal sac terminations were equally mid-L1 and S2, respectively. This suggests gender has a limited effect on the positions of the conus medullaris and thecal sac. However, the mean conus level across both genders was extremely similar, which disagrees with previous studies in adults [4] which found that gender has a small but significant impact on termination levels of the conus medullaris. However, studies amongst children [3] have equally demonstrated no significant difference across genders. This may suggest that gender may only become an influencing factor once adulthood is reached. This notion is strengthened in a prior study where women, particularly elderly aged ones, had a lower lying conus medullaris [13]. This may suggest that a more complex relationship between gender, age, and termination positions exists.

### Effect of age on the levels of termination of the conus medullaris and the thecal sac

Prior studies in children aged 1–6 months have found a mean termination position of upper L2 [3]. This study obtained a mean position of lower L1 (8.56) for the termination level of the CM in the first 6 months, suggesting variability exists. Furthermore, researchers have found that in 15–20-year-olds, the mean termination level was the middle third of L1 [3]. These studies implied that the conus may have been more low-lying in pediatric populations and may, in fact, ascend into adulthood, but that its termination position may gradually increase across increasing childhood age ranges and in fact further into adulthood.

No statistically significant differences were observed with increasing age on the final termination position of the conus medullaris within the first 6 months of life. This seems incongruent with the notion that the conus medullaris reaches its final adult position at 2 or 5 months old. Non-significant results were additionally obtained for increased age on the termination level of the conus medullaris across the entire pediatric population. This supports the idea that no further ascent occurs following childbirth and supports the notion that ascent is entirely in utero, between weeks 9 and 16 [14].

No statistically significant difference was noted amongst age ranges and the termination level of the thecal sac. These results are in agreement with the literature [4,15] that increasing age has no impact on the thecal sac level and this suggests no ascent occurs following childbirth.

### Limitations

The relative paucity of studies amongst the age group of 0-1 years of life is a limitation of this study. However, this is to be expected given routine spinal MRI in an otherwise asymptomatic infant is uncommon.

The assessment method employed in the study may have introduced observer bias between neuroradiologists, and this was minimised by consensus reads in equivocal cases.

Confounding factors such as child height were difficult to control, especially in tertiary referral centres catering to diverse communities where significant changes in pediatric height were observed.

## Conclusion

This study reaffirmed the fact that a Gaussian distribution exists with respect to the levels of termination of both the CM and TS within the pediatric population. Gender was shown to have a limited effect, and there was no general relationship between increasing age and termination levels, suggesting that the final level is obtained at birth.

Future work should be conducted to elucidate the relationship between increasing age on termination positions. This study highlighted the critical importance of imaging investigations in light of this Gaussian distribution and the knock-on effects they play in patient care.

## Supplementary information

ESM 1 (DOCX 300 kb)
