# Supplementary material for: Assessment of the levels of termination of the conus medullaris and thecal sac in the pediatric population
Source: Neuroradiology. 2023 Jan 21;65(4):835–43. doi: 10.1007/s00234-022-03111-8 (PMC10033476; doi:10.1007/s00234-022-03111-8)
Supplement: Supplementary file 1 — (DOCX 300 kb) [file 234_2022_3111_MOESM1_ESM.docx]

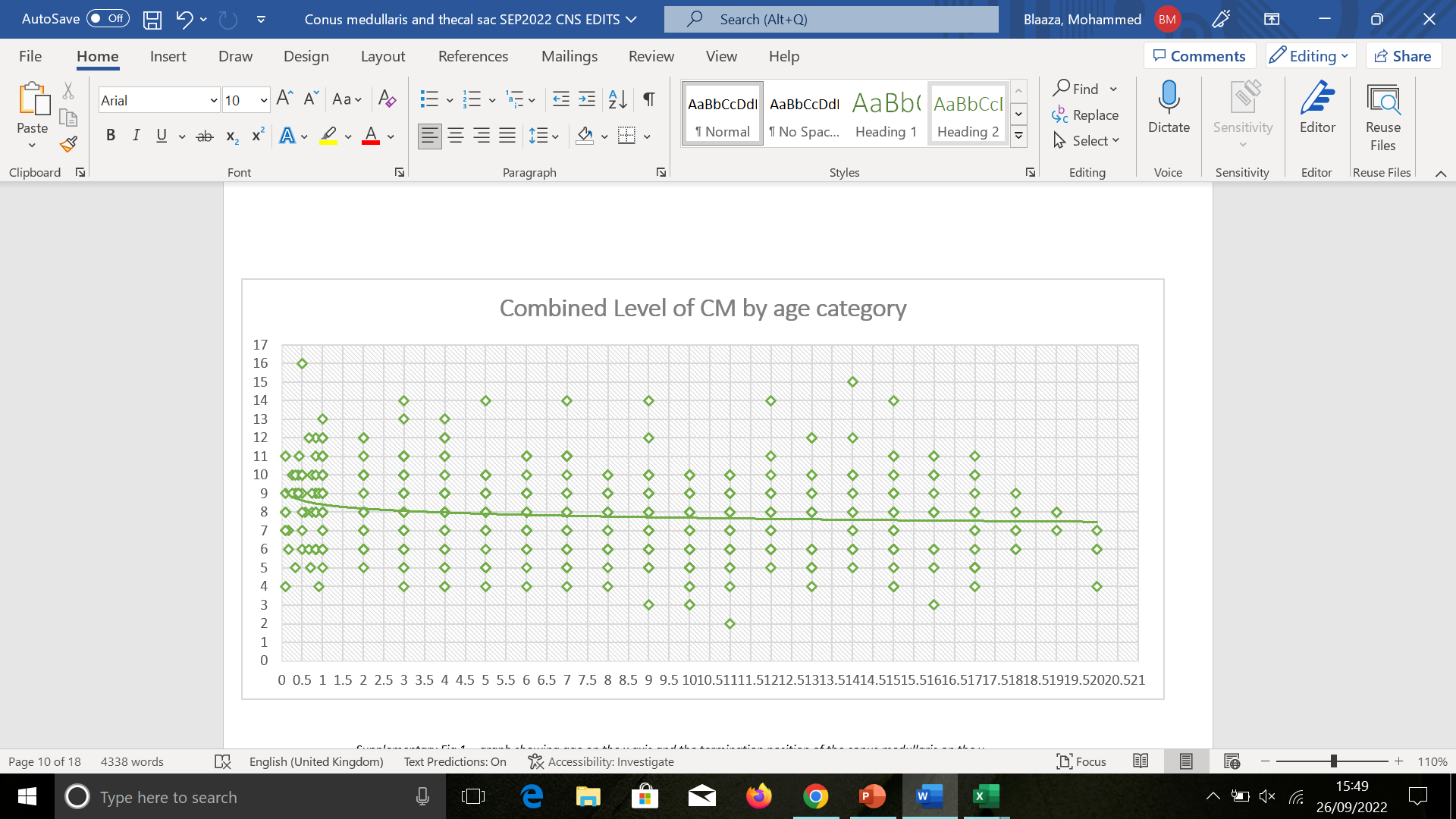


*Supplementary Fig 1 - graph showing age on the x axis and the termination position of the conus medullaris on the y axis where 1 is lower T11 and 16 is mid L3.*
